# T-cell responses to KSHV infection: a systematic approach

**DOI:** 10.18632/oncotarget.22683

**Published:** 2017-11-25

**Authors:** Romin Roshan, Nazzarena Labo, Matthew Trivett, Wendell Miley, Vickie Marshall, Lori Coren, Elena M. Cornejo Castro, Hannah Perez, Benjamin Holdridge, Eliza Davis, Rodrigo Matus-Nicodemos, Victor I. Ayala, Raymond Sowder, Kathleen M. Wyvill, Karen Aleman, Christine Fennessey, Jeffrey Lifson, Mark N. Polizzotto, Daniel Douek, Brandon Keele, Thomas S. Uldrick, Robert Yarchoan, Claes Ohlen, David Ott, Denise Whitby

**Affiliations:** ^1^ AIDS and Cancer Virus Program, Leidos Biomedical Research, Frederick National Laboratory for Cancer Research, Frederick, MD, USA; ^2^ Vaccine Research Center, National Institute of Allergy and Infectious Disease, Bethesda, MD, USA; ^3^ HIV and AIDS Malignancy Branch, National Cancer Institute, Bethesda, MD, USA

**Keywords:** KSHV, ELISpot, T-cells, cell-mediated immunity

## Abstract

Prior studies of T-cell responses to KSHV have included relatively few participants and focused on relatively few KSHV antigens. To provide a more comprehensive analysis, we investigated T-cell responses to the whole KSHV proteome using IFN-γ ELISpot. Using ∼7,500 overlapping 15mer peptides we generated one to three peptide pools for each of the 82 KSHV ORFs. IFN-γ ELISpot analysis of PBMCs from 19 patients with a history of KSHV-associated disease and 24 healthy donors (11 KSHV seropositive) detected widely varied responses. Fifty six of the 82 ORFs were recognized by at least one individual but there was little overlap between participants. Responses to at least one ORF pool were observed in all 19 patients and in 7 seropositive donors. Four seropositive donors and 10 seronegative donors had no detectable responses while 3 seronegative donors had weak responses to one ORF. Patients recognised more ORFs than the donors (p=0.04) but the response intensity (spot forming units: SFU per million cells) was similar in the two groups. In four of the responding donors, individual peptides eliciting the predominant responses were identified: three donors responded to only one peptide per ORF, while one recognized five. Using intracellular cytokine staining in four participant samples, we detected peptide-induced IFN-γ, MIP1-β, and TNF-α as well as CD107a degranulation, consistent with multifunctional effector responses in CD8+ and CD4+ T cells. Sequence analysis of TCRs present in peptide specific T-cell clones generated from two participants showed both mono- and multi-clonotypic responses. Finally, we molecularly cloned the KSHV specific TCRs and incorporated the sequences into retroviral vectors to transfer the specificities to fresh donor cells for additional studies. This study suggests that KSHV infected individuals respond to diverse KSHV antigens, consistent with a lack of shared immunodominance and establishes useful tools to facilitate KSHV immunology studies.

## INTRODUCTION

Kaposi’s sarcoma-associated herpesvirus (KSHV) is a gammaherpesvirus that establishes a prevalently latent and, in most individuals, asymptomatic lifelong infection [1]. KSHV also causes malignancies, including Kaposi’s sarcoma (KS)[2], and primary effusion lymphoma (PEL)[3]; as well as multicentric Castleman’s disease (MCD) a lymphoproliferative disorder [4]. The most common KSHV-associated disease is KS, which can occur in HIV uninfected people, especially older men in the Mediterranean (Classic KS); sub-Saharan Africans (endemic KS); and transplant recipients (iatrogenic KS) but most frequently develops in those with HIV infection (AIDS KS). Reduction of immunomodulatory therapy can result in regression of iatrogenic KS [5], and the introduction of combination antiretroviral therapy (cART) has dramatically reduced the incidence of AIDS-KS [6, 7]. The elevated risk of KS in immunocompromised hosts and the risk-reduction following recovery of T-cell function indicate that a loss of cell-mediated responses plays an important role in KS development. The critical role of T cells in maintaining control of chronic herpesviruses is demonstrated in the natural history of Epstein-Barr virus (EBV), human cytomegalovirus (HCMV) and herpes simplex virus (HSV)[8–10].

A loss of T-cell function is a major contributor to the development of AIDS-associated KS and iatrogenic KS. Consequently, the fundamental role of cell-mediated immunity in KSHV-associated pathogenesis has been apparent since the emergence of these clinico-pathological forms of KS. However, the study of KSHV-specific cell-associated immunity has progressed slowly, and more than 20 years after the identification of the virus, we have a remarkably limited understanding of the nature of cellular immune responses to infection. Indeed, there are relatively few studies of KSHV-specific cellular immunity; these have utilized varied methods, design, and scope, and the findings have rarely been replicated between studies making comparisons difficult [8]. Consequently, our understanding of cellular immune responses to KSHV is rudimentary, compared to the wealth of research available for EBV and HCMV. For HCMV, a comprehensive study in which overlapping peptides were synthesised across the entire proteome showed that most viral proteins are antigenic and that most infected individuals have CD4+ and CD8+ T cells that robustly recognise many different viral proteins [11] Partial data for KSHV suggests that a similarly variable range of proteins may elicit cellular immune responses, but compared to HCMV, it seems that infected individuals have relatively weak responses to only a few proteins [12]. The need to better understand KSHV-specific cellular immunity is becoming more urgent as KS is increasingly diagnosed in HIV infected persons with well-controlled HIV disease and robust CD4 cell counts [13, 14].

In a recent study, we examined antibody responses to the entire array of KSHV encoded proteins, the KSHV proteome. Using this unbiased systematic approach, we have shown that the antibody response to KSHV can be highly variable in both breadth and strength [15]. Here we use the same proteome-wide approach to investigate cellular immune response to KSHV. Using an interferon gamma (IFN-γ) ELISpot assay combined with both traditional and molecular immunological techniques, we identify cellular immune responses to KSHV and characterise the phenotype and functionality of the responding cells.

## RESULTS

### Participant characteristics

Four hundred and thirty-two RDP participants were tested for antibodies to KSHV using ELISA assays detecting anti-KSHV IgG against the lytic antigen K81 and the latent antigen LANA, encoded by ORF73. Thirty individuals (12.9%) who tested positive for either antigen on one or more occasions (up to 2903 days of follow up) were classified as KSHV seropositive, while the remainder were considered seronegative. Eleven KSHV seropositive individuals were available for this study; 13 seronegative individuals were also selected. The characteristics of the selected individuals are described in Supplementary Table 1. Nineteen HAMB patients with history of pathologically confirmed KSHV-associated diseases were recruited. All 19 were KSHV seropositive, and 15 of them were HIV co-infected. Healthy donors were significantly more likely to be female (p=0.0008) and white (p=0.005).

### KSHV viral load

KSHV DNA was detected in PBMCs of one healthy donor but the level was too low to be reliably quantified (qualitative positive: QP, Supplementary Table 1). Eight of 19 HAMB patients with a history of KS/PEL/MCD, had detectable KSHV DNA in PBMCs, two were QP and the remainder had viral loads ranging from 13-1000 copies per million PBMCs. KSHV viral load was not detected (<3copies/million cells) in PBMCs of the remaining subjects (Supplementary Table 1).

### Identification of T-cell responses to KSHV ORFs by ELISpot assay

**Figure 1:**
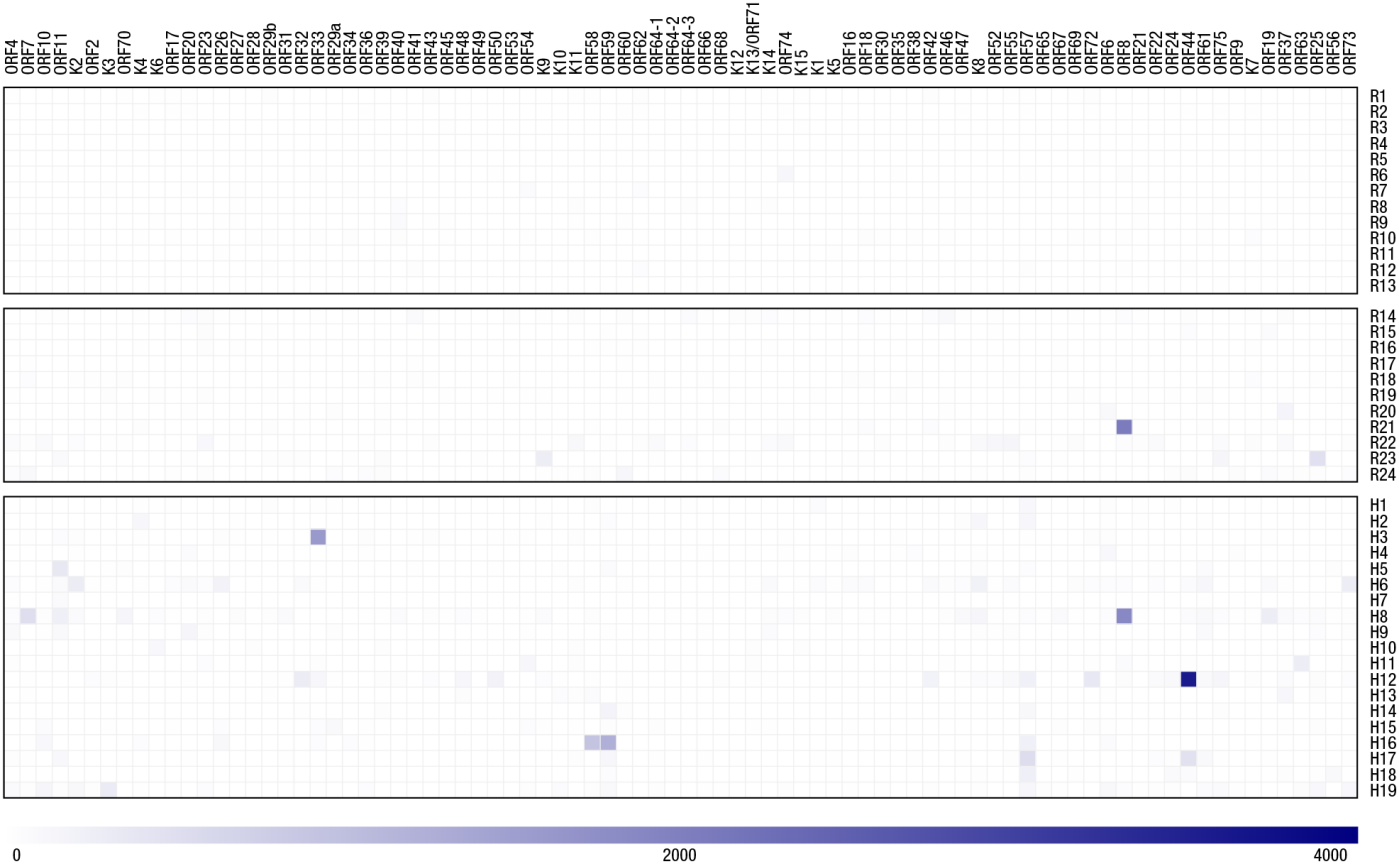
Summary of IFN-γ ELISpot responses to the KSHV proteome A heat map plot of IFN-γ ELISpot responses detected by our KSHV proteome analysis is presented with ORF pools examined in indicated in columns and donor codes identified in rows. Responses are denoted by blue-coloured squares with the intensity of each response proportional to the intensity of its corresponding square. A reference intensity scale is proved below the heat map.

Figure 1 and Supplementary Table 2 show the IFN-γ responses to 82 KSHV-encoded proteins measured in all participants using the ELISpot assay. Peptide pools representing 56 different ORFs (mostly lytic genes) elicited responses, and there was very little overlap between individuals regarding which antigens were recognized. No response to any ORF was seen in 10 of the KSHV seronegative healthy individuals. Three KSHV seronegative healthy individuals had weak responses to a single ORF. Four of the KSHV seropositive healthy donors had no response to any ORF while the remaining seven had responses to between 1 and 13 ORFs. All HAMB patients showed responses to between 1 and 17 ORFs.

The intensity of the responses was generally low: 49% of the responses were weak (60-100 SFU/10^6^ PBMCs) and 40% moderate (100-300 SFU/10^6^ PBMCs), while only 11% were strong responses (greater than 300 SFU/ 10^6^ PBMCs). In comparison, mean response intensities to the positive control pool of CMV, EBV and Influenza peptides were all moderate or strong (100-1000 SFU/10^6^ PBMCs) Mean responses were not significantly different in patients with KSHV-related diseases compared to healthy seropositive donors (HAMB: median 120, interquartile range (IQR) 92-245; RDP: median 92, IQR 82-249; p=0.5), but patients with KSHV related diseases recognized more antigens (median 3, IQR 2-6 vs. median 1, IQR 0-4, p=0.04). HLA typing results for all participants (Supplementary Table 3) revealed no association between HLA type and responses to specific antigens nor breadth/strength of overall response.

For the control wells, coefficients of variation were as follow: CEF, 1.17; SIV, 2.06, medium, 1.01. Distributions (median, IQR) of control responses did not differ between patients and donors, nor between seronegative and seropositive individuals, and were overall as follow: CEF, 300, 87, 1267; SIV, 0, 0, 7, medium 7,0, 13; 99^th^ percentiles were 54 and 40 for SIV and medium. Limited longitudinal ELISpot assays were performed when follow up samples were available (donors R6,7,9,14,20,21 and 23 with ORF8, ORF25, ORF37, ORF40, ORF41, ORF46, ORF54, ORF74), 2-5 timepoints were tested during a median interval of 273 days (IQR 34-384) Median inter assay CV was 0.28 (IQR 0.04-0.45).

### Peptide pool deconvolution and peptide identification

In order to identify the specific peptides eliciting cell-mediated responses in a given ORF peptide pool, an array matrix was used in four of the participants (R14, R20, R21, R23). In three of these participants, a single peptide per ORF pool (out of 88 to 274 comprising the pool) was identified. In one individual 5 peptides (out of a pool of 49) were identified. Peptide sequences are provided in Table 1.

**Table 1:** Peptides identified by deconvolution

	ORF	Number of peptides in pool	Number recognised	Sequence	T cell type
R14	ORF46	49	5	RLTTTVYPPQDKLMW	CD4+
R14	ORF46	49	5	VYPPQDKLMWWSHCC	CD4+
R14	ORF46	49	5	TGLAFSVDPQCQVPP	CD4+
R14	ORF46	49	5	SVDPQCQVPPSLRSI	CD4+
R14	ORF46	49	5	GVLLLNTVLTVEKGR	CD4+
R20	ORF37	96	1	KVYEIKCRFKYTFAK	CD4+
R21	ORF8	167	1	AKLASTHVPNGTVQY	CD4+
R23	ORF25	274	1	NKNNVPLCFGYQNAL	CD8+
R23	K9	88	1	LLRAGSDGDRATHHM	CD4+

### CD8+ and CD4+ specific ELISPOT assay

**Figure 2:**
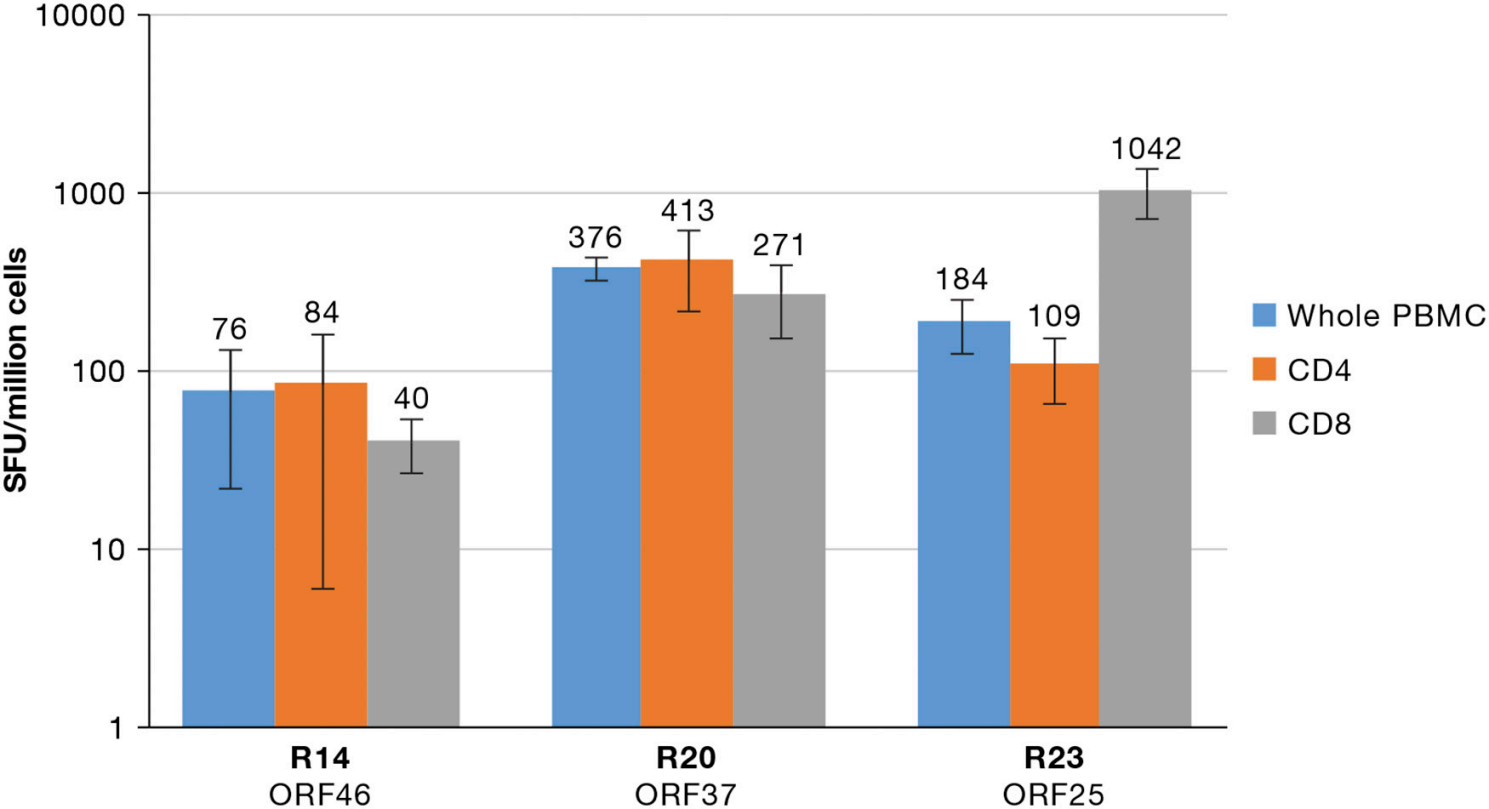
Presence of CD4+ and CD8+ specific IFN-γ ELISpot T-cell responses A graph of IFN-γ responses obtained by CD8^+^ and CD4^+^ sorting followed by type-specific ELISpot assay from three healthy donors is presented. The number of spots is presented on the*y*-axis and the donors and respective ORF pools under the *x*-axis.

In order to further delineate the responding T cells, CD4+ and CD8+ T-cell specific ELISpot assays were performed for participants R14, R20, and R23. All three donors displayed both CD4+ T-cell and CD8+ T-cell responses. Participants R14 and R20 had greater CD4+ T-cell responses while individual R23 had a greater CD8+ T-cell response (Figure 2).

### Multifunctional effector anti-KSHV T-cell responses

**Figure 3:**
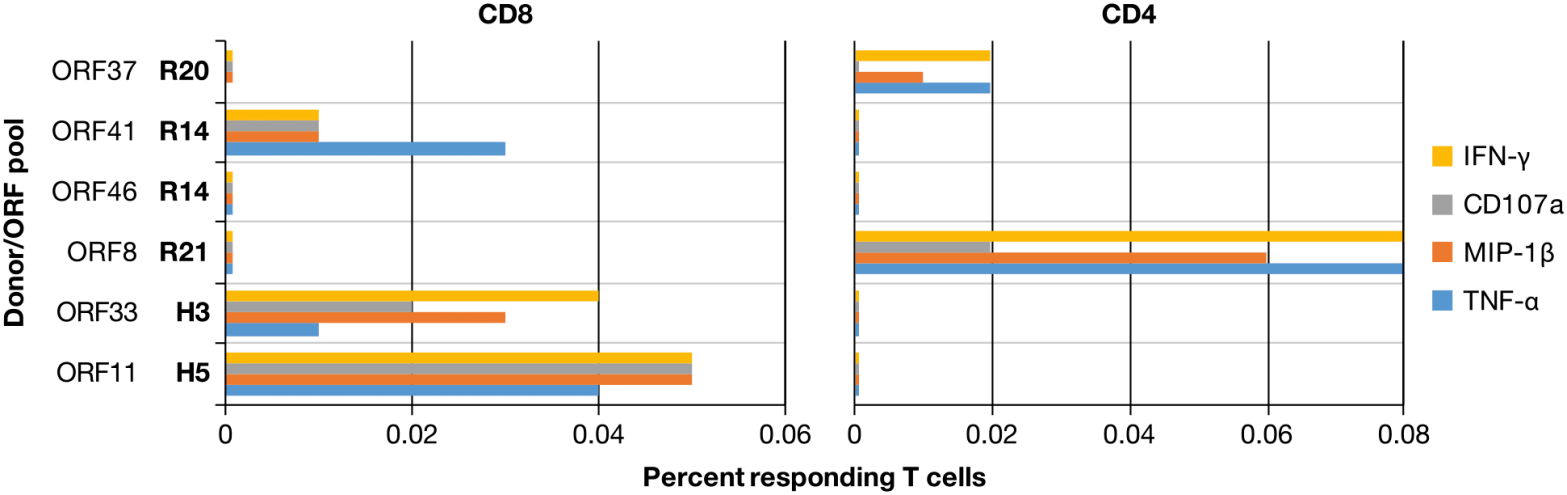
Multifunctional effector responses in donor PBMC A summary of flow cytometry for peptide-induced effector responses in PBMC from selected KSHV responding donors identified by whole proteome ELISpot analysis is presented. Donors and their respective ORFs are indicated on the *y*-axis and the percent of responding CD3^+^ cells out of >250,000 gated CD8^+^ or CD4^+^ T-cell events are displayed on the axis.

To confirm the ELISpot responses, induction of intracellular IFN-γ in T cells upon co-culture with PBMCs pulsed with their reactive ORF peptide pool was examined by flow cytometry. In PBMCs of 5 participants, intracellular flow cytometry detected antigen-peptide induction of IFN-γ in either CD8+ or CD4+ T cells (Figure 3). This is consistent with the results of the whole proteome IFN-γ ELISpot analysis except that donor R14 had a CD8+ response against ORF 41, but not ORF 46. Given the higher sensitivity of the ELISpot assay, this inability to detect a response to ORF 46 is likely due to the low frequency of responding cells in the PBMCs of this donor. The frequencies of IFN-γ responding cells were between 0.02-0.08%, consistent with the weak and moderate responses enumerated in the ELISpot assay. One hallmark of effector responses is the induction of multiple cytotoxic markers: intracellular IFN-γ, MIP-1β and TNF-α as well as surface CD107a, a component of cytolytic granules which appears upon degranulation. Flow cytometry using this panel revealed that in all of the IFN-γ producing T cells at least two additional markers could be detected with the majority having all four, consistent with polyfunctional effector responses.

### Analysis of KSHV-reactive T-cell clones

**Figure 4:**
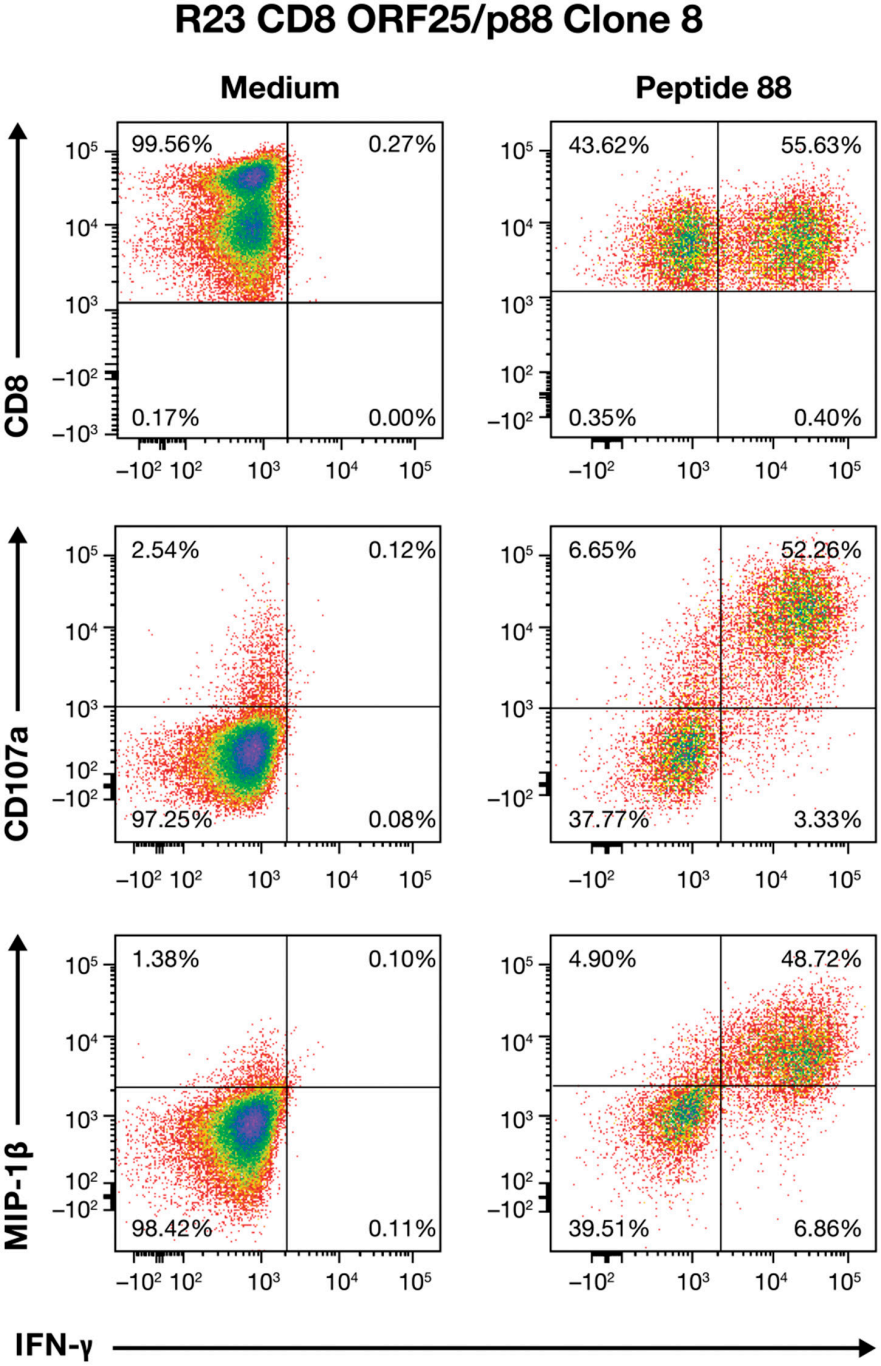
Multifunctional effector response profile of CD8+ ORF25/p88 Clone 8 T cells from donor R23 Flow cytometry analysis of ORF25-peptide 88-stimulated Clone 8^+^ T cells for IFN-γ, CD107a, and MIP-1β responses are presented.

While the results above show multifunctional responses in the reacting population of T cells, they do not indicate whether any individual T cell would have this characteristic. To address this issue, we isolated clones from peptide-pulsed PMBCs of healthy seropositive donors. Despite the low frequencies of responders in these donors, we successfully isolated a total of seven clones from two donors. Donor R23 yielded three clones reactive to the lytic ORF25 pool, peptide 88 (P88) as initially observed by ELISpot analysis and peptide deconvolution. R23 Clone 8, a CD8+ T-cell clone, was further examined for multifunctional responses. Flow cytometry analysis revealed that the fluorescence intensity of intracellular IFN-γ staining in the reacting R23 Clone 8 T cells was directly proportional to CD107a and MIP-1β intensity, indicating the presence of polyfunctional effector T cells (Figure 4). In contrast, the TNF-α response was rather low and was not investigated further. The robust and coordinated production of these two classic effector responses indicate the presence of a functional antiviral CD8+ T-cell response in this donor.

**Figure 5:**
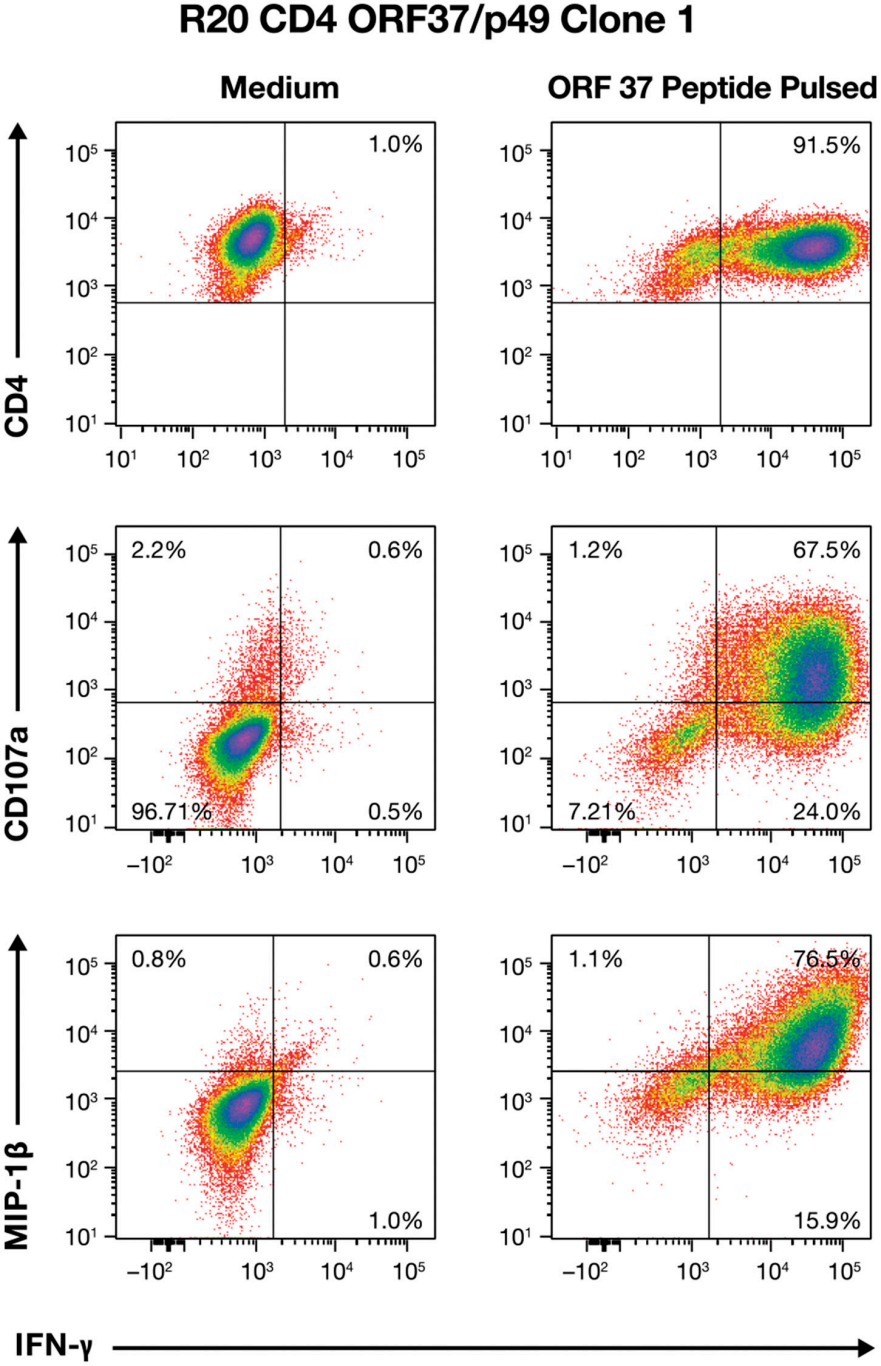
Multifunctional effector response profile of CD4+ ORF37/p49 Clone 1 T cells from donor R20 Flow cytometry analysis from ORF37/peptide 49-stimulated Clone 1 T cells for IFN-γ, CD107a, and MIP-1β responses are presented.

Donor R20 had CD4+ T cells responding to the lytic ORF 37, peptide 49 (P49) and yielded 4 clones. Flow cytometry analysis of R20 Clone 1 showed that, like in R23 Clone 8, both CD107a and MIP-1β responses were highly correlated with IFN-γ production, indicating that R20, Clone 1 also displayed an authentic CD4+ effector response (Figure 5). Similar results were obtained for the other three R20 clones (data not shown).

### Clonotypic analysis of responding T-cell clones

**Figure 6:**
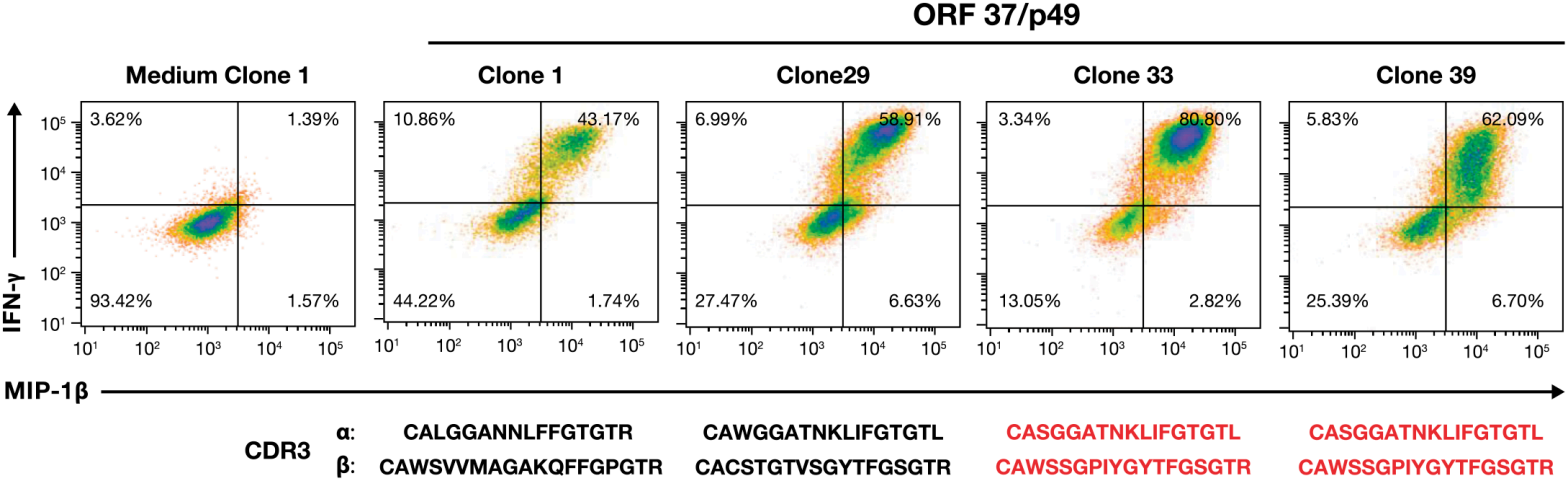
Induced cytokine response profiles and TCR clonotype analysis of ORF37 T-cell clones IFN-γ, and MIP-1β flow cytometry analysis of four ORF37/peptide 49-stimulated CD4^+^ T-cell clones are presented with their α and β CDR3 sequences displayed under their corresponding dot plots. Shared CDR3 sequences are indicated in red.

To determine whether these clones were monotypic, i.e. T cells that originated from a single clonal expansion in the donor, or were the result of independently formed and selected clones, we sequenced the complementary determining region (CDR3) of both the α and β T-cell receptor (TCR) genes, representing the products of unique recombination events which determine receptor specificity and antigen affinity. The results revealed three unique CDR3 sequences from the four R20 ORF 37 responding CD4+ T-cell clones with Clones 1 and 29 being unique and 33 and 39 sharing the same sequence (Figure 6). Therefore, we detect the presence of at least three clonotypes, each representing an independent recombination/selection/expansion event. In contrast, clonotypic analysis of clones from R23 showed that Clones 1, 2 and 8, responding to ORF25, P88, all shared the same CDR3 sequence α: CAVLDSNYQLIWG and β: CASSILGLRNTEAFF. Therefore, they likely represent a single clonotypic response that arose from an expansion of a single clone.

### Transfer of ORF37 TCR

**Figure 7:**
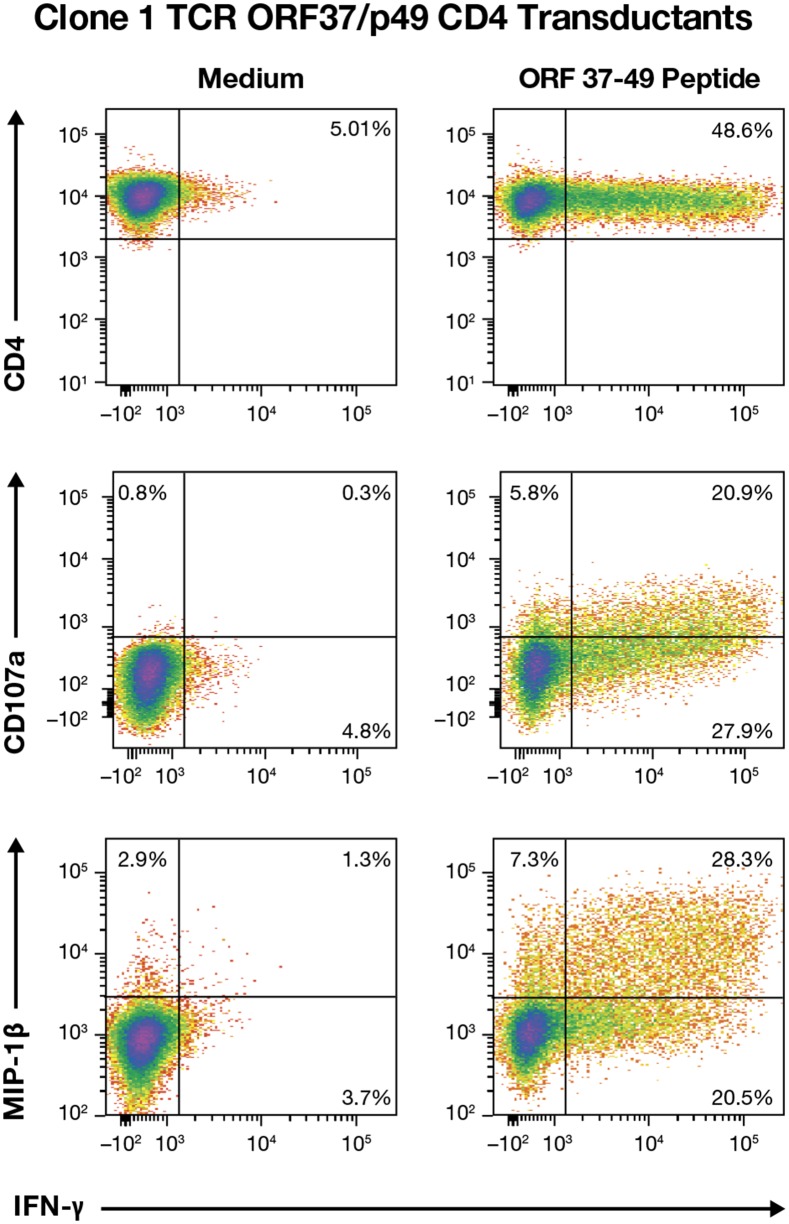
Multifunctional effector responses from Clone 1 TCR transductions IFN-γ, CD107a, and MIP-1β flow cytometry for analysis of donor R20 CD4^+^ T cells transduced with the ORF37/p49 TCR that were stimulated with the ORF37-peptide 49 are presented.

The immunological study of T cells is hindered by their limited life-span, which makes comparison between clones obtained at different times or by different investigators nearly impossible. CD8+ T cells isolated from PMBC typically have a 5-7 months maximum life-span before they senesce and die, and CD4+ T cells persist in culture for a shorter time, typically 4-6 months maximum (MTT unpublished data). To preserve TCR specificities of interest for additional studies we have developed a method to clone TCRs from T cells into a murine retrovirus-based TCR transfer vector to place the TCRs onto other MHC restriction-matched cells [16–21]. To capture the ORF37/p49 response, we cloned the Vα and Vβ regions of TCRs from R 20 Clones 1, 29, and 33 directly into a TCR transfer vector with PCR primers reverse engineered from the CDR3 sequence analysis [18]. Since no tetramer is available for the ORF37 p49 specificity, we genetically marked transduced cells by co-expressing a C-terminally truncated non-signaling p75 low affinity nerve growth factor receptor gene (tNGFR) [18, 22]. Transduction of fresh CD4 T cells from donor R20 conveyed TCR specificity for the ORF37/p49 peptide in sorted NGFR^+^ T cells for all three TCRs, which also exhibited antigen-induced CD107a degranulation and both IFN-γ and MIP-1β responses (representative data for Clone 1 presented in Figure 7).

**Figure 8:**
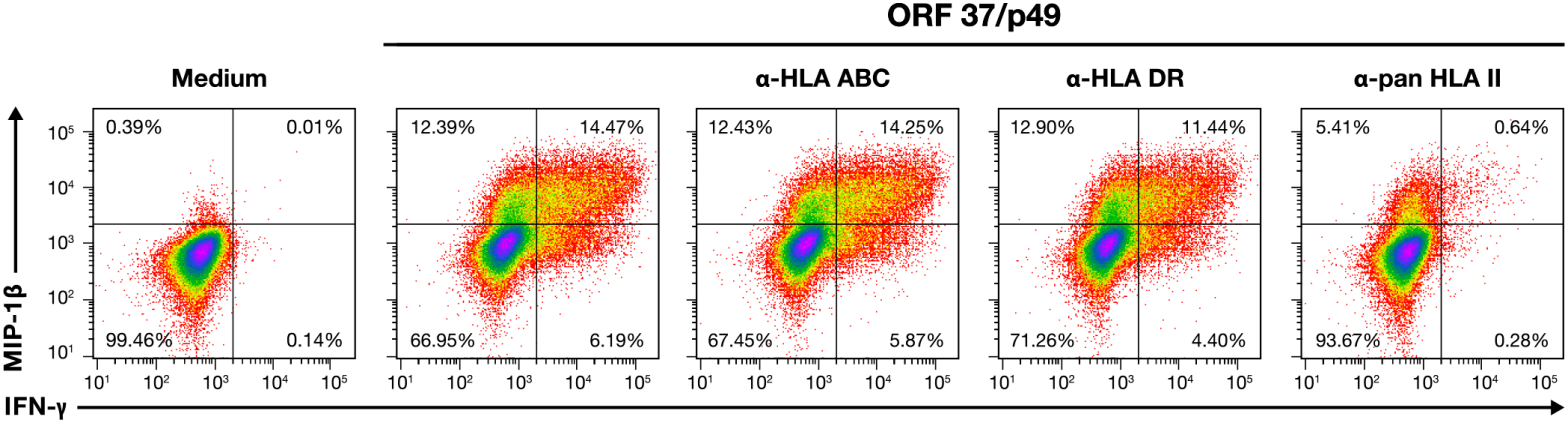
MHC restriction of ORF37/p49 TCR IFN-γ, and MIP-1β flow cytometry results for cognate peptide induced responses from CD4^+^ T cells transduced with the Clone 1 ORF37/peptide 49 TCR in the presence of HLA-blocking antibodies are presented. Treatment of each culture is indicated above their respective dot plots.

While an increasing range of functional phenotypes has been identified for CD4+ T-cells, they are classically identified with indirect helper functions rather than effectors with a direct virus-suppressing role. Although CD4 T cells with an effector response have been documented, they are somewhat unusual. To assess the MHC restriction of the CD4+ TCRs, we performed a series of HLA-blocking experiments. Since the original clones had senesced and died, we recreated this specificity by using donor R20 CD4 T cell TCR transductants for the blocking experiments. Clone 1 and 33 transductants were stimulated with ORF37-p49 in the presence of a series of HLA class 1 and 2 antibodies known to block antigen presentation; blocking was measured by a reduction in the induced IFN-γ response. As expected, the HLA-ABC class I blocking antibody failed to reduce the IFN-γ response (21%). However, the levels of the IFN-γ response greatly decreased from 21 to 1% in the presence of the pan-MHC-II blocking antibody (Figure 8). In contrast, with the HLA-DR blocking antibody the IFN-γ response had a negligible decrease to16%. Therefore, this TCR appears to be class II-restricted with either DP or DQ, rather than DR as the restricting HLA element. Sequencing indicated either HLA-DP^*^04:02 or 15:01; DQA^*^05 or 01:02; DQB^*^13:05 or 15:03 as possible restricting alleles.

## DISCUSSION

We present here a systematic approach to the study of cellular immune responses to KSHV. ELISpot analysis using a library of peptides covering the entire KSHV proteome revealed no immunodominant INF-γ responses to KSHV antigens. In seropositive, healthy donors and in patients with KSHV-related diseases, responses were diverse and sparse with most responders recognizing between one and five ORFs. The intensity of responses was modest overall (∼90% of responses were under 300 SFU/million PBMCs) and similar in seropositive healthy donors and patients, while the breadth of the responses was wider in patients than in seropositive donors. The diversity of the individual responses observed and the lack of shared immunodominance perhaps helps explain the diverse observations reported in previous limited studies of cellular immune responses to KSHV [23–28]. The paucity of KSHV responding cells may also result in variability due to sampling within individuals and may also contribute to the lack of consensus reported in the literature. The low intensity of response is consistent with presumably latent infection in many of our participants, as corroborated by their undetectable or low PBMC viral load. Nevertheless, the frequency of responders identified by ICS in healthy individuals appeared to be roughly 10% of that typically reported for other herpesviruses such as EBV [29] and HCMV [11]. The reasons for such sparsity and the mechanism of such economical, successful viral control merit further investigation.

We observed weak responses in 3 of the KSHV seronegative participants, consistent with some previous studies that included seronegative participants [12, 30]. This is perhaps not surprising in healthy donors from a low KSHV prevalence region, whose antibody levels are expected to be low, resulting in some misclassification of infection status. In all participants, the majority of the responses were to proteins involved in the lytic stage rather than the latent phase of the viral life cycle, consistent with previous reports [12]. This could be due to the dynamics of KSHV initial infection and the generation of the T-cell response. In this scenario, initial responses that were likely raised soon after infection and lytic viral replication would lead to memory T cells produced against lytic proteins as we observe. It is also possible that intermittent and/or localized pockets of low-level lytic replication maintain the low but definite T-cell responses against lytic antigens.

The INF-γ responding cells displayed a cytolytic effector profile: flow cytometry showed peptide induced production of MIP-1β and TNFα and CD107 degranulation, which identified these cells as authentic effector T cells. Polyfunctional responses to KSHV have also been reported elsewhere [31, 32]. Such responses have been reported to be associated with HIV control and non-progression in HIV disease [33, 34]. In this study both CD8+ and CD4+ antiviral effector T-cell responses were detected. The presence of CD4+ T cells with direct effector responses, while unusual, has been described previously. Indeed, several cytolytic T-cell responses to viruses inducing chronic disease, including the herpesviruses EBV and human cytomegalovirus are predominantly from CD4+ T-cell clones [35]. CD4+ T cells are also directly implicated in the control of both HIV and SIV [16, 36–39].

Molecular analysis of T-cell clones isolated from healthy seropositive donors R20 and R23, found differences in the breath of the responses. CDR3 sequencing of four ORF37, p49-specific CD4+ T-cell clones isolated from donor R20 demonstrated three unique clonotypes, suggesting that this response had at least three unique naïve T cells that were independently selected and expanded by a separate interaction with the same KSHV antigen. In contrast, three ORF25, p 88-reactive CD8+ T-cell clones from donor R23, shared the same peptide and had the same clonotype, thus representing a single clone that had been expanded by a KSHV antigen interaction in this individual. We could therefore define the ORF25/p88 response observed in donor R23 as narrow and the ORF37/p49 response as broader. This study presents the first such clonotypic analysis for KSHV and while the significance of these results is not clear, they do show that the T-cell specificities generated to a KSHV epitope can be variable.

Our initial attempt to use clones to study T-cell responses to KSHV were severely hindered by the short life spans of these cells, dying before they can be analyzed (MTT, VIA, CO and DW unpublished observations). Here, we successfully extended our analysis of KSHV-specific T-cell clones past their rather short life-span. We transferred two of the three R20 ORF37/p49 specificities to fresh R20 donor CD4+ T cells and confirmed HLA class 2 restriction, narrowing the possibilities to six DP or DQ alleles. We are currently identifying the restricting elements to enable experiments using cells from other compatible donors and these TCRs of interest.

While we observed responses to synthesized peptides in our study, we did not study responses to other kinds of KSHV antigens such as whole viral lysates or recombinant proteins. It is possible that native epitopes might not be presented by HLA due to the inability of the cell to correctly process such antigens. Current efforts are focusing on developing antigen-expressing cells to confirm our results with authentic peptide presentation. We were also unable to address the possible effects of KSHV strain variation on T cell responses. Specifically, because our peptides were designed based on the BC-1 cell line, divergent KSHV strains may not recognize all of these peptides. In general, KSHV strains are likely to be highly conserved across most of the ORFs tested and while whole genome sequencing of KSHV using next generation sequencing (NGS) approaches may allow the study of this in future, the role of genetic variation in KSHV T cell immunity is beyond the scope of the current study. In addition, current NGS approaches for whole genome KSHV sequencing depend on samples with much higher viral loads than were observed in our participants and so would not have been feasible in the current study.

In summary, we present here the development of an in-depth comprehensive strategy to identify and capture in an unbiased manner, T-cell responses to KSHV in infected individuals using both traditional and molecular immunology techniques. This strategy provides a pathway to better understand the natural history of KSHV infection and the pathogenesis of KSHV associated malignancies. Much of this proof- of-principle analysis has been focused on healthy recallable donors partly to ensure availability of additional material for the isolation and testing of T-cell clones. We are currently using this strategy to better define the T-cell response in more patients with KSHV-associated diseases. We hope to better define the relationship between these responses and disease outcomes, and to elucidate the potential correlates of protection from KSHV associated diseases.

## MATERIALS AND METHODS

### Study participants

Twenty-four healthy donors were recruited from the Frederick National Laboratory for Cancer Research (FNLCR), Research Donor Program (RDP), which enrolls scientists and support personnel working in the facility. Nineteen patients with KSHV-related diseases were recruited from the HIV and AIDS Malignancy Branch (HAMB) clinic of the National Cancer Institute (NCI). All participants were enrolled in study protocols approved by the relevant National Cancer Institute Institutional Review Board. All gave written informed consent in accordance with the Declaration of Helsinki.

### KSHV serology

KSHV ORF73 and K8.1 ELISA were performed as previously described [40]. Briefly, ELISA plates were coated with KSHV recombinant proteins ORF 73 and K8.1. Plasma or serum samples were diluted 1:20 for K8.1 and 1:100 for ORF 73 and incubated in antigen coated plates at 37°C for 1.5 hours. Captured antibodies were detected using a phosphatase labelled goat anti-Human IgG antibody (KPL, Gaithersburg, MD) and the 1-step PNPP substrate (Thermo Scientific, Waltham, MA). Optical density was measured using the Spectramax plus 384 spectrophotometer.

### KSHV peptides: synthesis and preparation

Approximately 7,500 peptides were synthesised as 15mers overlapping by 10 amino acids across the entire KSHV genome using SynPhase Lantern technology (Mimotopes, Victoria, Australia). Peptide sequences were based on the sequence of BC-1 cell line derived virus [41]. Eighty four peptide pools were made, 81 corresponding to a single ORF and 3 for the very large ORF 64. Individual lyophilised peptides were reconstituted using 50% acetonitrile. Peptide pools were prepared by combining reconstituted peptides for each ORF and were frozen and re-lyophilised. Lyophilised pools were reconstituted in DMSO (< 20%) and PBS. Each pool comprised 10-274 peptides, at a concentration of 5ug/ml/peptide.

### Quantitative PCR

KSHV viral load was determined using a previously described method [42]. Briefly, DNA was extracted from peripheral blood mononuclear cells (PBMCs) and KSHV viral load per million cells was quantified by real time PCR using primers for the KSHV K6 gene and the human ERV-3 [43].

### HLA typing

Genotyping of 7 HLA loci (HLA-A, -B, -C, -DQA1, -DQB1, -DRB1 and -DPB1) was performed by PCR-sequence-specific oligonucleotide probing and PCR-sequence-based typing.

### Enzyme-linked immunospot assay

For each participant, one IFN-γ pre-coated 96 well ELISpot plate (Mabtech, Cincinnati, U.S.A) was used to assess responses to the 82 KSHV ORFs. Two positive controls: anti-human CD3 and a CMV, EBV, Influenza peptide pool (CEF, AnaSpec, Fremont, U.S.A) and two negative controls: medium alone and SIV Gag CM9 peptide (New England peptide, Gardner, U.S.A) were used. All controls were plated in triplicates while the KSHV ORFs were plated in single wells.

The anti IFN-γ 96 well ELISpot plate was seeded with 150,000 freshly processed PBMCs, or sorted T cells per well and incubated for 18 hours at 37°C, 5% CO2. One-step detection with mAb, 7-B6-1 conjugated to ALP developed with BCIP/NBT-plus substrate was used and read using the CTL ImmunoSpot Analyzer (Cellular Technology Limited, Shaker Heights, USA). Wells with spot counts of 60 spot forming units (SFU) per million PBMCs or greater were considered positive, based on the distribution of responses in negative and positive controls. Responses were confirmed in follow up samples when possible (donors R6,7,9,14,20,21 and 23).

### CD8+ and CD4+ specific ELISpot

CD3+ CD4+ T cells and CD3+ CD8+ T cells were positively isolated from PBMCs using the MACS separation columns with CD4 microbeads or CD8 microbeads (Miltenyi biotec, Auburn, U.S.A) according to manufacturer’s instructions. CD8+ and CD4+ specific ELISpot assays were performed as described above.

### Peptide deconvolution

To identify the reacting peptide(s) within an ORF pool, a peptide pool deconvolution array method was used. Donors were selected based on availability of follow up samples and ORFs were selected based on the results of the initial assay. Peptides from a single KSHV ORF were plated in partial pools (at 5ug/ml/peptide) using a deconvolution matrix [44]. Partial pools were arranged within the matrix so that every peptide was present in two pools allowing for the identification of individual peptide/peptides eliciting responses by INF-γ ELISpot by shared reactivity in the matrix. Reactivity of individual peptides was confirmed by INF-γ ELISpot assay.

### Flow cytometry and intracellular cytokine staining

Flow cytometry was carried out as previously described [44]. The following antibodies were used for surface staining: anti-CD3 (SP34-2), antiCD45 (HI30), anti-CD4 (OKT4), anti-CD8 (SK1), anti-CD28 (CD28.2), anti-CD107a (H4A3), anti- IFN-γ (B27), anti-macrophage inflammatory protein 1 beta (MIP-1β; D21-1351), anti-TNF-α (Mab11) [BD Biosciences]. This was followed by surface staining for CD3, CD4, CD8, and/or anti-low affinity nerve growth factor receptor (L-NGFR; ME20.4-1.H4) (Miltenyi Biotech). Subsequently, the cells were fixed and permeabilized followed by intracellular staining for IFN-γ, TNF-α and MIP-1β. Samples were acquired promptly on a BD LSRII or Fortessa flow cytometer (BD biosciences). More than 250,000 either CD4+or CD8+ T cells per sample were acquired and data were analyzed using FCS Express software (DeNovo). Data summarised are from analysis of at least 250,000 CD8+ or CD4+ gated T cells in the PBMC culture with results subtracted for background observed in matching unstimulated cultures.

### T-cell culture

T cells were cultured in RPMI 1640 with 10% fetal bovine serum, 100 IU/ml penicillin/streptomycin, and 100 IUml IL-2 (Peprotech). T-cell cultures were expanded and maintained by stimulation by coculture with a mixture of irradiated human PBMCs (4x10^6^/mL), purified anti-CD3 antibody (60ng/mL) and purified CD28 antibody (60ng/mL) (both from BD Biosciences).

### IFN- γ selection

KSHV reactive T-cells were magnetically selected for IFN-γ secretion using the IFN-γ secretion assay Cell Enrichment and Detection Kit (Miltenyi Biotec) per manufacturer instructions. In brief, peptide stimulated cells were labeled with an IFN-γ capture antibody immediately after a 6 hour stimulation with peptide pulsed autologous cells. The labeled cells were allowed to rest for 45 minutes at 37°C in complete media with frequent resuspension to allow capture of secreted IFN-γ on the surface. This was followed with a second anti-IFN-γ antibody labeled with PE and subsequent sorting using anti PE microbeads and an LS column/magnet apparatus. Typical enrichment was 10-50 fold with final antigen specific frequencies of 50-80%.

### Generation of T-cell clones by limiting dilution cloning

Fresh PBMCs or PBMCs thawed and rested overnight at 37°C were enriched for antigen specific cells by co-culture with peptide-pulsed autologous PBMC for 7 days for a total of three times as previously described [16]. The T-cell cultures were enriched 2-3 times and T cells with KSHV IFN-γ secretion responses were selected from the amplified cultures using the IFN-γ secretion enrichment. Typical enrichment was 10-50 fold with final antigen specific frequencies of 50-80%. Antigen-responding T cells from the enriched cultures were cloned by limiting dilution as previously described [17].

### TCR clonotype analysis

A portion of the TCR α and β variable regions encompassing the complementary determining region 3 (CDR3) was amplified using a nested set of primers, one for the α chain and one for the β chain. For each set, the 5’ primer consisted of a pool of optimized sequences covering the entire α or β variable segments. The 3’ primer consisted of a set of single antisense α or β constant primers V-region cDNA was generated from RNA from T-cell clones using an outside primer set and the following amplification cocktail: 5μl 2X Super Script III/ Platinum Taq buffer (cat # 11753-100, ThermoFisher Scientific), 5μl of the 2.5μM (total primer concentration) α/β 5’-3’ outside primer mix, 0.1 μl SUPERase inhibitor (cat # AM2694, ThermoFisher Scientific), 0.1 μl RNAse OUT (cat # 10777019, ThermoFisher Scientific), 1 μl Super Script III/ Platinum Taq, 1.4-X μl cell PCR grade water, and cells for a final volume of 10 μl. Reactions were carried out in a thermocycler using a single tube RT-PCR program (50°C, 15 min; 19 cycles: 95°C, 15 s; 60°C, 4 min; then 4°C hold. The cDNA was then diluted 1:1 with PCR grade water and then used in two separate 2^nd^ round PCR reactions using either the α or β inside nested primer set using the following amplification cocktail: 4.5μl first round RT-PCR reaction, 2.5μl 10X HotStar Taq buffer (cat # 203605, Qiagen), 0.5 μl dNTP (cat. #, Bio-39053 Bioline), 5μl 5X Q solution (Qiagen), 1.5 μl each of both the 10 μM (total primer concentration) α or β 5’ inside primer and α or β 3’ inside primer mix, 0.17 μl HotStar Taq (cat # 203605, Qiagen), and 9.3 μl water for a final volume of 25 μl. and amplified with a 95°C, 15 s; 51 cycles:

94°C, 15 s; 50°C, 30 s, 72°C, 60 s; 72°C, 10 min, then 4°C hold thermocycler program. Products were purified and cloned using the Topo-XL cloning kit (cat. # K4750-20, ThermoFisher Scientific) and sequenced to obtain the CDR3 sequences for both α and β chains. The results for Clone 39 were verified by RNASeq, using the TruSeq Stranded RNA LT kit and the MiSeq platform (Illumina).

### TCR transfer

The partial V-region sequences produced from the CDR3 analyses were used to bioinformatically develop PCR primers to the 5’ sequence of the V-gene sequences of the ORF37/p49 TCRs Clones 1, 29, and 33 isolated from donor R20 and amplify and clone the V-regions from cDNA into a TCR transfer vector, HuTCR-NGFR, as previously described [18]. TCR vector constructs were transfected into the Phoenix-RD114 vector packaging line [45] to produce TCR transfer vectors which were then used to transduce primary R20 donor T cells using spinoculation onto vector-loaded, RetroNectin (Takara Bio USA)-coated plates according to the provider’s instructions as previously described [19]. Transductants were observed by flow cytometry of the coexpressed truncated, non-signaling NGFR proteins on the cell surface using phycoerythrin-conjugated anti-L-NGFR (Miltenyi Biotech) staining and isolated by L-NFGR immunoaffinity sorting using Miltenyi Biotech anti-PE microbeads.

### HLA-class restriction of isolated TCRs

HLA-blocking experiments of IFN-γ intracellular cytokine induction assays were used to determine the class restriction of the ORF37, peptide 49 TCRs. CD4+ T-cells from donor R20 transduced with either ORF 37/p49 R20 Clone 1 or R20 Clone 33 TCRs were used in an IFN-γ intracellular cytokine induction assay using R20 donor PBMCs as targets. To facilitate gating of effector cells, the target T-cells were first stained with CellTraceViolet (Thermo/Invitrogen) according to the provider’s protocol. Labelled R20 PBMC targets were blocked with HLA-A,-B,-C antibody (Clone W6/32, Biolegend, 311402), HLA-DR antibody (Clone G46-6, BD Biosciences, 555810), or HLA-DR, DP, DQ antibody (Clone TU39, BD Biosciences, 555557) for 30 min at room temperature. The cells were washed with 7 volumes of medium to remove any unbound blocking antibody then pulsed with 1μg/mL ORF 37/p49 peptide for 30 min at 37°C followed by a wash to remove any unbound antigen peptide. The blocked and untreated targets were then used in an otherwise routine IFN-γ intracellular assay with the TCR transductant lines.

### Statistical analysis

Two-sample Wilcoxon rank-sum (Mann-Whitney) test were used to compare demographic variables, number of responses and mean response intensity. All analyses were performed using Stata v13.1.

## SUPPLEMENTARY MATERIALS TABLES








