# Supplementary material for: T-cell responses to KSHV infection: a systematic approach
Source: Oncotarget. 2017 Nov 25;8(65):109402–16. doi: 10.18632/oncotarget.22683 (PMC5752530; doi:10.18632/oncotarget.22683)
Supplement: Supplementary file 1 [file oncotarget-08-109402-s001.pdf]

# T-cell responses to KSHV infection: a systematic approach

## SUPPLEMENTARY MATERIALS

**Supplementary Table 1: Summary characteristics of study participants**

See Supplementary File 1

**Supplementary Table 2: HLA genotypes of study participants**

See Supplementary File 2

**Supplementary Table 3: Elispot Count data corresponding to Figure 1**

See Supplementary File 3
