# Supplementary material for: T-cell responses to KSHV infection: a systematic approach
Source: Oncotarget. 2017 Nov 25;8(65):109402–16. doi: 10.18632/oncotarget.22683 (PMC5752530; doi:10.18632/oncotarget.22683)
Supplement: Supplementary file 2 [file oncotarget-08-109402-s002.docx]

**Supplementary Table S1**. **Summary characteristics of study participants.**

| **ID** | **Age** | **Sex** | **Ethnicity** | **Geography** | **KSHV serology** | **KSHV Viral load in PBMC^a^** | **HIV status** | **Disease^b^** |
| --- | --- | --- | --- | --- | --- | --- | --- | --- |
| R1 | 32 | F | White | unknown | — | 0 | — | n/a |
| R2 | 38 | M | White | unknown. | — | 0 | — | n/a |
| R3 | 65 | M | White | unknown | — | 0 | — | n/a |
| R4 | 55 | F | White | unknown | — | 0 | — | n/a |
| R5 | 68 | F | White | unknown | — | 0 | — | n/a |
| R6 | 50 | F | White | unknown | — | 0 | — | n/a |
| R7 | 59 | F | White | unknown | — | 0 | — | n/a |
| R8 | 54 | F | White | unknown | — | 0 | — | n/a |
| R9 | 50 | F | White | unknown | — | 0 | — | n/a |
| R10 | 34 | F | White | unknown | — | 0 | — | n/a |
| R11 | 25 | M | White | unknown | — | 0 | — | n/a |
| R12 | 33 | M | White | unknown | + | 0 | — | n/a |
| R13 | 29 | M | White | unknown | + | 0 | — | n/a |
| R14 | 44 | M | White | unknown | + | 0 | — | n/a |
| R15 | 65 | M | White | unknown | + | 0 | — | n/a |
| R16 | 37 | M | White | unknown | + | 0 | — | n/a |
| R17 | 45 | F | Native American | unknown | + | 0 | — | n/a |
| R18 | 47 | M | White | unknown | + | 0 | — | n/a |
| R19 | 56 | F | Black | unknown | + | 0 | — | n/a |
| R20 | 31 | F | Hispanic | unknown | + | 0 | — | n/a |
| R21 | 47 | F | White | unknown | + | 0 | — | n/a |
| R22 | 50 | M | White | unknown | + | 0 | — | n/a |
| R23 | 36 | F | White | unknown | + | 0 | — | n/a |
| R24 | 53 | M | White | unknown | + | QP | — | n/a |
| H1 | 69 | M | White | Middle East | + | 0 | — | KS |
| H2 | 55 | M | Black | N. America | + | 0 | + | KS |
| H3 | 60 | F | Black | East Africa | + | 0 | + | History of KS+MCD |
| H4 | 67 | M | White | Middle East | + | 287 | — | History of KS |
| H5 | 60 | M | White | Middle East | + | 0 | — | KS |
| H6 | 29 | M | Black | N. America | + | 0 | + | History of MCD+ PEL |
| H7 | 53 | M | White | N. America | + | 0 | + | History of KS |
| H8 | 59 | M | White | N. America | + | 0 | + | History of PEL |
| H9 | 29 | M | unknown | N. America | + | 0 | + | KS/PEL |
| H10 | 38 | M | Black | N. America | + | 13 | + | KS |
| H11 | 38 | M | Asian | W. Pacific | + | 0 | + | KS |
| H12 | 56 | M | White | N. America | + | QP | + | KS |
| H13 | 60 | M | White | N. America | + | 0 | + | KS/MCD |
| H14 | 84 | M | White | S.E. Europe | + | 160 | — | KS |
| H15 | 30 | M | Black | N. America | + | 0 | + | KS |
| H16 | 41 | M | Black | W. Africa | + | 42 | + | KS/KICS |
| H17 | 27 | M | White | N. America | + | 93 | + | KS |
| H18 | 52 | M | Black | N. America | + | QP | + | KS |
| H19 | 41 | M | Black | N. America | + | 0 | + | KS |

^a^viral load expressed as genome equivalents/10^6^ cells or QP, qualitative positive

^b^KS, Kaposi’s sarcoma; MCD, multicentric Castleman’s disease; PEL, primary effusion lymphoma; n/a, not applicable,
