# Supplementary material for: T-cell responses to KSHV infection: a systematic approach
Source: Oncotarget. 2017 Nov 25;8(65):109402–16. doi: 10.18632/oncotarget.22683 (PMC5752530; doi:10.18632/oncotarget.22683)
Supplement: Supplementary file 3 [file oncotarget-08-109402-s003.docx]

**Supplementary Table 2. HLA genotypes of study participants**

| **ID** | **A** | | **B** | | **C** | | **DPB1** | | **DQA1** | | **DQB1** | | **DRB1** | |
| --- | --- | --- | --- | --- | --- | --- | --- | --- | --- | --- | --- | --- | --- | --- |
| R1 | 03 | 29 | 07 | 49 | 07 | 07 | 02 | 04 | 03 | 01 | 03 | 06 | 04 | 15 |
| R2 | 01 | 03 | 27 | 57 | 02 | 07 | 03 | 04 | 01 | 01 | 06 | 06 | 13 | 15 |
| R3 | 01 | 11 | 51 | 58 | 07 | 15 | 03 | 03 | 04 | 01 | 04 | 05 | 08 | 14 |
| R4 | 02 | 33 | 44 | 58 | 03 | 05 | 02 | 04 | 03 | 01 | 03 | 06 | 04 | 13 |
| R5 | 02 | 25 | 15 | 15 | 01 | 03 | 04 | 13 | 03 | 01 | 03 | 06 | 04 | 13 |
| R6 | 02 | 02 | 07 | 44 | 05 | 07 | 03 | 04 | 03 | 01 | 03 | 06 | 04 | 15 |
| R7 | 02 | 03 | 15 | 35 | 04 | 07 | 03 | 04 | ND | ND | 03 | 06 | 11 | 13 |
| R8 | 24 | 32 | 44 | 44 | 02 | 05 | ND | ND | ND | ND | ND | ND | ND | ND |
| R9 | 01 | 25 | 08 | 18 | 07 | 12 | 04 | 10 | 01 | 01 | 05 | 05 | 01 | 14 |
| R10 | 02 | 26 | 51 | 57 | 01 | 06 | 02 | 04 | 03 | 05 | 03 | 03 | 04 | 11 |
| R11 | 02 | 03 | 15 | 15 | 03 | 03 | 03 | 03 | 03 | 03 | 03 | 03 | 04 | 04 |
| R12 | 02 | 11 | 08 | 44 | 04 | 07 | 02 | 04 | 03 | 05 | 03 | 03 | 04 | 11 |
| R13 | 03 | 03 | 38 | 44 | 12 | 16 | 02 | 02 | 03 | 02 | 03 | 02 | 04 | 07 |
| R14 | 23 | 68 | 35 | 38 | 04 | 04 | 03 | 04 | 05 | 01 | 02 | 06 | 03 | 15 |
| R15 | 03 | 30 | 07 | 18 | 05 | 07 | 03 | 03 | 01 | 01 | 05 | 06 | 01 | 15 |
| R16 | 01 | 03 | 13 | 27 | 02 | 06 | 04 | 17 | 02 | 01 | 02 | 06 | 07 | 15 |
| R17 | 02 | 11 | 07 | 39 | 07 | 07 | 04 | 04 | 04 | 01 | 04 | 06 | 08 | 15 |
| R18 | 03 | 30 | 13 | 35 | 04 | 06 | 03 | 04 | 03 | 02 | 03 | 02 | 04 | 07 |
| R19 | 24 | 30 | 44 | 57 | 02 | 18 | 04 | 04 | 01 | 01 | 05 | 05 | 01 | 01 |
| R20 | 24 | 31 | 14 | 38 | 08 | 12 | 04 | 15 | 05 | 01 | 03 | 06 | 13 | 15 |
| R21 | 02 | 24 | 44 | 51 | 05 | 16 | 04 | 04 | 03 | 01 | 03 | 06 | 04 | 15 |
| R22 | 01 | 02 | 08 | 15 | 03 | 07 | 04 | 04 | 01 | 03 | 06 | 03 | 03 | 04 |
| R23 | 01 | 11 | 08 | 35 | 04 | 07 | 01 | 20 | 05 | 01 | 02 | 06 | 03 | 13 |
| H1 | 11 | 24 | 40 | 52 | 03 | 14 | ND | ND | ND | ND | 03 | 03 | 04 | 11 |
| H2 | 74 | 74 | 49 | 57 | 07 | 18 | ND | ND | ND | ND | 03 | 05 | 01 | 13 |
| H3 | 23 | 68 | 14 | 57 | 07 | 08 | ND | ND | ND | ND | 05 | 05 | 0. | 12 |
| H4 | 03 | 24 | 08 | 35 | 04 | 07 | ND | ND | ND | ND | 03 | 03 | 04 | 11 |
| H5 | 01 | 02 | 14 | 39 | 06 | 07 | ND | ND | ND | ND | 05 | 05 | 01 | 14 |
| H6 | 03 | 74 | 58 | 58 | 06 | 06 | ND | ND | ND | ND | 02 | 06 | 07 | 11 |
| H7 | 01 | 23 | 15 | 44 | 02 | 07 | ND | ND | ND | ND | 02 | 06 | 07 | 13 |
| H8 | 03 | 31 | 07 | 35 | 04 | 07 | ND | ND | ND | ND | 05 | 06 | 01 | 13 |
| H9 | 02 | 32 | 15 | 40 | 03 | 03 | ND | ND | ND | ND | 06 | 06 | 13 | 15 |
| H10 | 02 | 24 | 41 | 44 | 02 | 17 | 03 | 04 | 05 | 05 | 03 | 03 | 11 | 13 |
| H11 | 11 | 24 | 15 | 48 | 07 | 08 | 05 | 05 | 05 | 01 | 03 | 05 | 11 | 15 |
| H12 | 02 | 69 | 15 | 18 | 05 | 07 | 01 | 10 | 05 | 01 | 02 | 06 | 03 | 13 |
| H13 | 02 | 24 | 41 | 44 | 02 | 17 | ND | ND | ND | ND | 03 | 03 | 11 | 13 |
| H14 | 03 | 24 | 39 | 51 | 05 | 12 | 04 | 04 | 05 | 01 | 03 | 06 | 11 | 15 |
| H15 | 02 | 02 | 42 | 53 | 04 | 17 | 02 | 02 | 04 | 01 | 04 | 06 | 03 | 15 |
| H16 | 02 | 30 | 42 | 58 | 07 | 17 | ND | ND | ND | ND | 03 | 04 | 03 | 11 |
| H17 | 01 | 02 | 18 | 52 | 07 | 12 | ND | ND | ND | ND | 03 | 05 | 11 | 16 |
| H18 | 02 | 30 | 18 | 57 | 05 | 18 | 02 | 02 | 05 | 01 | 02 | 05 | 03 | 13 |
| H19 | 02 | 68 | 07 | 37 | 02 | 03 | ND | ND | ND | ND | 02 | 03 | 07 | 13 |
